# Epidemiology and mortality of *Clostridium difficile* infection: a 5-year retrospective laboratory-based study in a large teaching hospital in Northern Italy

**DOI:** 10.1186/2047-2994-4-S1-P25

**Published:** 2015-06-16

**Authors:** A Orsi, C Alicino, V Faccio, M Zacconi, F Ansaldi, P Durando, G Icardi

**Affiliations:** 1 University of Genoa, Genoa, Italy

## Introduction

*Clostridium difficile* represents the major infective causes of hospital-acquired infectious diarrhoea and one of the most common microorganisms responsible for health-care associated infection.

## Objectives

To describe incidence and mortality associated with CDI in a large northern Italy teaching hospital in a 5-year period and to analyze the association between risk factors in patients with CDI and 30-days all-causes mortality.

## Methods

We performed a retrospective study at IRCCS AOU San Martino – IST, a 1,300-beds tertiary adult acute-care teaching hospital in Genoa, Italy. Between 1 January 2010 and 31 December 2014, true numbers of hospital patient-days were obtained from the hospital digital archives of patients’ clinical charts. Numbers of CDI were identified through the computerized microbiology laboratory database. Demographic, administrative and clinical data were collected for all patients included in the analysis. Annual incidence rates of CDI were calculated as the number of events per 10,000 patient-days. In addition, annual incidences of CDI were stratified according to the ward where the diagnosis of CDI was made. Finally, 30-day survivals of CDI patients were estimated and univariate and multivariate analysis was performed to identify independent risk factors associated with 30-days all-causes mortality.

## Results

Between January 2010 and December 2014, we identified 388 episodes of CDI. The overall incidence of CDI during the five-years study period was 0.61/10,000 patient-days. The incidence steadily increased during the five year study period reaching the peak of 3.04/10,000 patient-days in 2014. The highest annual incidences were registered in rehabilitation units, with a peak of 10.01 CDI/10,000 patient-days in 2013. Thirty-days all-causes mortality was 26,1% and the main risk factors, independently predicting mortality, were increasing age and pathological level of albumin and creatinine at the diagnosis of CDI.

## Conclusion

In our hospital we observed a marked increase in the incidence of CDI during the five-years study period. More efforts might be necessary to control the diffusion of *Clostridium difficile* in our hospital, because of the increasing incidence and the high mortality rate of related infections.

## Disclosure of interest

None declared.

